# Draft Genome Sequence of *Streptomyces specialis* Type Strain GW41-1564 (DSM 41924)

**DOI:** 10.1128/genomeA.00101-17

**Published:** 2017-03-30

**Authors:** Lotfi Loucif, Caroline Michelle, Jérôme Terras, Jean-Marc Rolain, Didier Raoult, Pierre-Edouard Fournier

**Affiliations:** a Unité de Recherche sur les Maladies Infectieuses et Tropicales Émergentes (URMITE), UM 63 CNRS 7278, IRD 198, INSERM 1095, IHU Méditerranée Infection, Aix-Marseille-Université, Marseille, France; b Laboratoire de Biotechnologie des Molécules Bioactives et de la Physiopathologie Cellulaire (LBMBPC), Faculté Des Sciences de la Nature et de la Vie, Université de Batna 2, Batna, Algeria

## Abstract

Here, we report the draft genome sequence of *Streptomyces specialis* type strain GW41-1564, which was isolated from soil. This 5.87-Mb genome exhibits a high G+C content of 72.72% and contains 5,486 protein-coding genes.

## GENOME ANNOUNCEMENT

The genus *Streptomyces* was proposed in 1943 by Waksman and Henrici (1). This genus currently contains more than 780 species with validly published names, which makes it the largest bacterial genus (2, 3). In 2008, Kämpfer et al. described the new species *S. specialis* with strain GW41-1564 as the type strain (2, 4). This Gram-positive, non-endospore-forming bacterium was isolated from soil. It exhibits an unusual quinone system, with the predominant compounds being MK-10(H_4_) and MK-10(H_6_) (4). Here, we report the draft genome sequence of *S. specialis* type strain GW41-1564 (= CSUR P2100 = DSM 41924).

The genomic DNA (gDNA) of *S. specialis* strain GW41-1564^T^ was sequenced using a paired-end strategy and the MiSeq Illumina sequencer (Illumina Inc, San Diego, CA, USA). The paired-end Nextera library was constructed from 1 ng of fragmented gDNA and loaded on two flow cells. The library was barcoded in order to be mixed with 12 or 15 other projects and sequenced using a 2 × 250-bp format. The sequencing produced a total of 4,096,640 paired-end reads.

The obtained Illumina reads were assembled using the SPAdes software (http://bioinf.spbau.ru/spades) (5). Open reading frames (ORFs) were predicted using Prodigal (6). The predicted bacterial protein sequences were searched using BLASTp against the Clusters of Orthologous Groups (COG) and the GenBank databases. tRNA gene detection was performed using the tRNAScanSE tool (7), whereas ribosomal RNA genes were predicted using RNAmmer (8).

The genome from *S. specialis* strain GW41-1564^T^ is 5,874,245 bp in length with a G+C content of 72.72%. After assembly, the genome is composed of 143 scaffolds (composed of 168 contigs). Of the 5,550 predicted genes, 64 were RNAs (three complete rRNA operons, two additional 5S rRNAs, and 53 tRNAs) and 5,486 were protein-coding genes. A total of 3,786 genes were assigned a putative function, and 306 genes were identified as ORFans. The remaining genes were annotated as hypothetical proteins (1,268 genes). This strain has four antibiotic resistance genes, and 59 genes were predicted as nonribosomal peptide synthases or polyketide synthases. Twenty-one bacteriocin-encoding genes were also identified in this genome.

### Accession number(s).

The genome sequence of *Streptomyces specialis* strain GW41-1564^T^ was deposited in GenBank under the accession number FAXE01000000.
